# Sex differences in the associations between adiposity distribution and cardiometabolic risk factors in overweight or obese individuals: a cross-sectional study

**DOI:** 10.1186/s12889-021-11316-4

**Published:** 2021-06-26

**Authors:** Yide Yang, Ming Xie, Shuqian Yuan, Yuan Zeng, Yanhui Dong, Zhenghe Wang, Qiu Xiao, Bin Dong, Jun Ma, Jie Hu

**Affiliations:** 1 Key Laboratory of Molecular Epidemiology of Hunan Province, School of Medicine, Hunan Normal University, Changsha, 410006 China; 2 Department of Child and Adolescent Health, School of Medicine, Hunan Normal University, Changsha, 410006 China; 3 Institute of Child and Adolescent Health, School of Public Health, Peking University Health Science Center, Beijing, 100191 China; 4 Department of Epidemiology, School of Public Health, Southern Medical University, Guangzhou, 510515 Guangdong China; 5 College of Information Science and Engineering, Hunan Normal University, Changsha, 410081 China; 6 Menzies Health Institute Queensland, Griffith University, Brisbane, Queensland 4111 Australia

**Keywords:** Fat distribution, Cardiometabolic health, Sex difference, Overweight and obesity

## Abstract

**Background:**

We aimed to assess the associations between adiposity distribution and cardiometabolic risk factors among overweight and obese adults in China, and to demonstrate the sex differences in these associations.

**Methods:**

A total of 1221 participants (455 males and 766 females) were included in this study. Percentage of body fat (PBF) of the whole body and regional areas, including arm, thigh, trunk, android, and gynoid, were measured by the dual-energy X-ray absorptiometry method. Central adiposity was measured by waist circumference. Clustered cardiometabolic risk was defined as the presence of two or more of the six cardiometabolic risk factors, namely, high triglyceride, low high density lipoprotein, elevated glucose, elevated blood pressure, elevated high sensitivity C-reactive protein, and low adiponectin. Linear regression models and multivariate logistic regression models were used to assess the associations between whole body or regional PBF and cardiometabolic risk factors.

**Results:**

In females, except arm adiposity, other regional fat (thigh, trunk, android, gynoid) and whole-body PBF are significantly associated with clustered cardiometabolic risk, adjusting for age, smoking, alcohol drinking, physical activity, and whole-body PBF. One-SD increase in Z scores of the thigh and gynoid PBF were significantly associated with 80 and 78% lower odds of clustered cardiometabolic risk (OR: 0.20, 95%CI: 0.12–0.35 and OR: 0.22, 95%CI: 0.12–0.41). Trunk, android and whole-body PBF were significantly associated with higher odds of clustered risk with OR of 1.90 (95%CI:1.02–3.55), 2.91 (95%CI: 1.75–4.85), and 2.01 (95%CI: 1.47–2.76), respectively. While in males, one-SD increase in the thigh and gynoid PBF are associated with 94% (OR: 0.06, 95%CI: 0.02–0.23) and 83% lower odds (OR: 0.17, 95%CI: 0.05–0.57) of clustered cardiometabolic risk, respectively. Android and whole-body PBF were associated with higher odds of clustered cardiometabolic risk (OR: 3.39, 95%CI: 1.42–8.09 and OR: 2.45, 95%CI: 1.53–3.92), but the association for trunk PBF was not statistically significant (OR: 1.16, 95%CI: 0.42–3.19).

**Conclusions:**

Adiposity distribution plays an important role in the clustered cardiometabolic risk in participants with overweight and obese and sex differences were observed in these associations. In general, central obesity (measured by android PBF) could be the best anthropometric measurement for screening people at risk for CVD risk factors for both men and women. Upper body fat tends to be more detrimental to cardiometabolic health in women than in men, whereas lower body fat is relatively more protective in men than in women.

**Supplementary Information:**

The online version contains supplementary material available at 10.1186/s12889-021-11316-4.

## Background

Obesity is a major public health issue in China and all over the world [1]. Although Asia has reportedly the lowest rate of obesity and overweight, there is an alarmingly increasing trend in the past decades, with the prevalence of overweight and obesity increasing from 12.6% in 1980 to 30.5% in 2015 [2, 3]. Asian people accumulate a higher percentage of body fat compared to Caucasians with the same body mass index (BMI) [2, 4]. Notably, China has the biggest number of affected people of overweight and obesity worldwide, in which nearly half (46%) of adults and 15% of children were overweight or obese [5]. Obesity has been recognized as a strong predictor of many cardiovascular diseases leading to a serious disease burden [6]. Particularly, obesity is closely related to dyslipidemia, elevated blood pressure (BP), impaired fasting glucose, chronic inflammation status and metabolic syndrome (MetS) [7–10]. Moreover, long term overweight and obesity may lead up to various non-communicable diseases, such as cardiovascular disease and cancer [11–13].

Public health implications of obesity could vary by sex and ethnicity. For example, the Danish prospective Inter99 study prospectively found that metabolic healthy obesity (MHO) was associated with a higher risk of ischemic heart disease (IHD) in male participants when comparing to metabolic healthy normal weight (MHN) participants but not found in females [14]. While, the China Kadoorie Biobank cohort study showed that MHO was significantly associated with a higher risk of IHD in both males and females [15]. The difference could be partially attributed to the different adiposity distribution between women and men in the Asian and Western population [16].

Significant sex differences were observed in adiposity distribution [17, 18] and the prevalence of, as well as the underlying mechanisms for cardiovascular health outcomes [19]. However, there is inconsistency in the findings of sex difference in the associations between adiposity distribution and cardiometabolic health outcomes [19–21]. This study examined the interactive effects between sex and regional fat distribution on the cardiometabolic risk factors. Exploration of the gender difference in fat distribution on cardiometabolic risk factors could help us understand the implications of the sex-specific mechanism for cardiometabolic outcomes, and consequently provide us valuable insights for sex-specific interventions on cardiometabolic risk factors.

Obesity indicates excessive body adiposity accumulation, which is defined by whole-body weight gain or high BMI. Notably, adiposity distribution also plays an important role in adverse cardiometabolic outcomes from childhood to adulthood [22–25]. Compared to their Western counterparts, Asian people are likely to experience a higher risk of cardiometabolic diseases at the same level of adiposity measured by BMI [26, 27]. Previous studies in the Chinese population used waist circumference [28], BMI [29] or bioelectrical impedance [30] to measure adiposity or fat distribution, which are not as accurate as Dual-energy X-ray absorptiometry (DXA, golden measurement for adiposity). DXA is a precise and direct measure used globally to evaluate the fat-free mass and fat mass of the full body and specific areas including arm, thigh, trunk, android and gynoid. It is a recommended method for visceral adiposity measurement due to the simplicity to use and minimal dose of radiation [31, 32]. The DXA method has been widely used in epidemiological studies to quantify the regional adiposity distribution.

Among the studies explored the associations between DXA-measured fat distribution and cardiometabolic indicators in Chinese population [33–40], participants were children in two studies [34, 40], women only in two studies [37, 39], and middle-aged women and men with a mean age over 46 years in four studies [33, 35, 36, 38]. While body composition or fat distribution varies substantially with age [41], limited studies had examined the associations of adiposity distribution and cardiometabolic risk among Chinese young adults.

Furthermore, previous work had focused on two to four metabolic syndrome components, whereas the relationship of fat distribution with clustered cardiometabolic risk factors or inflammatory cytokine is not comprehensively demonstrated [35, 36]. In the present study, we included a comprehensive profile of cardiometabolic risk factors, which were two lipid indicators (high triglyceride [TG] and low high-density lipids cholesterol [HDL]), two blood pressure (BP) indicators (systolic and diastolic BP), one blood glucose indicator (fasting glucose) and two inflammatory indicators (high sensitivity C-reactive protein, hs-CRP and adiponectin, ADI) respectively. Elevated C-reactive protein (CRP) and anti-inflammatory adipokines are both important inflammatory biomarkers for cardiovascular diseases [42–44]. For example, Wu et al. have revealed favorable associations of DXA-measured leg fat with ADI, BP, glucose, TG, HDL in both genders, but not for CRP, and unfavorable associations of trunk fat with CRP, ADI, BP in both genders and glucose only in men [33]. While, Snijder’s study reported opposite association of trunk and leg fat with glucose in both men and women [45]. The findings of this study may add novel information regarding the associations by sex between adiposity distribution and these inflammatory factors.

All in all, the present study aimed to examine the associations between DXA-measured adiposity distribution and cardiometabolic outcomes and its clustering, as well as to explore the sex differences in their relationships. We hypothesized the associations between regional fat distribution and cardiometabolic risk factors and its clustering differed in females and males.

## Methods

### Study population

Participants were recruited using a convenience sampling method in local urban communities of Haidian District, Beijing, China in 2014. Informed consents were sought from all participants before participating in the study. Details of data collection were published in our previous paper [46]. The inclusion criteria included: 1) has lived in Beijing for at least 1 year, and 2) was between 22 to 55 years old, and 3) was overweight (24 kg/m^2^ ≤ BMI < 28 kg/m^2^) or obese (BMI ≥ 28 kg/m^2^) [47]. A standard physical examination and medical history investigation was then conducted by trained clinical doctors to the eligible participants. Participants who have diseases related to important organs (such as heart, liver, kidney, or lung), physical deformities, or who self-reported with secondary obesity (clinically diagnosed obesity with a specific endocrine or genetic origin, such as Cushing’s syndrome, Prader-Labhard–Willi syndrome), were subsequently excluded. Besides, participants who were taking medicines, such as hypoglycemic agents, lipid-lowering drugs and antihypertensive drugs, were also excluded. Finally, a total of 1221 out of 1488 recruited participants who met the inclusion and exclusion criteria were included for the final analysis.

### Measurements

#### Anthropometric measurements

Anthropometric indicators (height, weight, and waist circumference) were measured by trained investigators with a standardized protocol. Participants were weighed at least twice to the nearest 0.1 kg using a standard lever scale (regularly calibrated with a counterpoise of 20 kg), with barefoot and light clothing. Height was measured at least twice to the nearest 0.1 cm (regularly calibrated) using a stadiometer. If the difference between repeated measurements was larger than 0.1 kg for weight or 0.1 cm for height, then further measurement(s) were conducted until the difference was within 0.1 kg or 0.1 cm. The mean value of the final two weight and height measurements were used for the final analysis. Waist circumference was measured with a Myotape scale (Accufitness, Green Villge, Colordo, USA) by trained investigators at the midpoint between the iliac crest and lowest rib, twice measurements of waist circumference were conducted to the nearest 0.1 cm, and the average value of twice measurements were used for data analysis.

#### BP measurements

We used a standard clinical sphygmomanometer to measure BP after the participants had been resting for at least 5-min. Two BP measurements for every participant with minimum 5 min time interval were required, and the measurement error was set as not more than 10 mmHg. If the difference between the two measurements was over 10 mmHg, BP was repeatedly measured, and the mean value of the final two measurements was used for analysis.

#### Measurement of body adiposity

BMI is the most commonly used measure of body adiposity, however, it provides an inaccurate evaluation of the body fat without assessing the body fat distribution. Alternatively, the DXA method is a golden standard for body fat measurement, which demonstrates a strong capacity in predicting fat distribution and its potential associations with the cardiometabolic risk factors [31]. The whole body and regional body compositions were measured by DXA scans with a Lunar iDXAME scanner (GE Healthcare, Lunar iDXAME + 210,205, America) in a hospital. The examination was conducted by experienced clinicians following standard procedures. Percentage of body fat (PBF) was used for analysis. Key parameters, of interest, included the whole body PBF and regional (Arm, trunk, thigh, android and gynoid) PBF. The arm area includes the arms and shoulder areas from the crease of the axilla to the glenohumeral joint. The trunk area vertically includes the area from below the chin to the femoral necks, the neck part, the chest part, the abdominal area and the pelvic area. The thigh area is comprised of the areas below the lower border of the trunk area. The android area is around the waist areas, is comprised of areas from the top of the pelvis (at iliac crests) up to 20% of the distance between the femoral neck and the pelvis. The gynoid area’s upper and lower boundaries are located below the iliac crest by 1.5 times and twice times the height of the android area [39]. Total PBF was defined as the ratio between total body fat mass and total body mass (including fat mass and fat-free mass). Regional PBF was defined as the ratio between regional fat mass and regional mass. For example, the arm PBF was defined as the ratio of the arm fat mass and arm mass.

#### Measurement of plasma biomarkers

Venous blood samples of participants were collected after 8 h of fasting. Serum high sensitivity C-reactive protein (hsCRP), blood lipids concentration and fasting glucose were measured immediately by the immune-turbidimetric assay (Automatic biochemical analyzer AU400, OLYMPUS, Japan). Serum concentrations of ADI were measured with an ELISA test using an enzyme standard analyzer (Enzyme analyzer model: DNX-9620A computer washer) with reagents of Human Total Adiponectin Immunoassay (R&D Systems, America).

##### Diagnostic criteria of metabolic syndrome (MetS), cardiometabolic risk factors and its clustering

In the present study, cardiometabolic risk factors included six components: impaired fasting glucose, elevated blood pressure, elevated TG, low HDL, elevated hsCRP and low ADI. The clustered cardiometabolic risk was defined as the presence of at least two of the six aforementioned components. The International Diabetes Federation Task Force has recommenced the following definitions of the five components of MetS [48]: 1) central obesity identified by waist circumference (female > 80 cm and male > 85 cm); 2) fasting blood glucose ≥5.6 mmol/ L or taking medicine for diabetes; 3) systolic blood pressure (SBP) ≥130 mmHg or diastolic blood pressure (DBP) ≥ 85 mmHg, or taking medicine for hypertension; 4) fasting triglyceride (TG) ≥ 1.7 mmol/L; 5) high-density lipids cholesterol (HDL) < 1 mmol/L for male or < 1.3 mmol/L for female. Additionally, for inflammatory biomarkers, elevated hsCRP was defined as hsCRP > 30 mg/L [49]; low ADI was defined as ADI ≤ 3.27 mg/L, and high ADI as ADI > 3.27 mg/L [50].

#### Measurement of covariates

Physical activity, sedentary time, age, smoking, and alcohol drinking were investigated through a questionnaire by face-to-face interview. Physical activities and sedentary time were investigated by the International Physical Activity Questionnaire-Short Form (IPAQ-SF) [51]. Four types of activities were investigated, namely vigorous physical activity, moderate physical activity, walking and sitting. Time (minutes) for each of the above activities were investigated, and utilized metabolic equivalent(MET) for each physical activity was calculated by multiplying the MET with time spent on this activity. Then, the utilized MET for the week was calculated by multiplying with the number of days the particular physical activity. Therefore, MET-min/week was obtained. For calculating MET-min/week for each participant, the values of MET used for our study was developed by the American College of Sports Medicine. For vigorous physical activity, moderate physical activity, walking and sitting behavior, the estimates were 8, 4, 3.3 and 1.5 METs, respectively. Alcohol drinking was categorized as drinking or not drinking in the past week. Similarly, smoking was categorized as smoking and not smoking in the past week.

### Statistical analysis

To estimate and compare the associations between a variety of fat distribution variables and cardiometabolic indicators, we calculated Z scores for WC and regional PBF using the following formula: Z score = (the original value-mean)/standard deviation. G*Power version 3.1.9.6 software was used to conduct the sample size estimation. Two-tailed alpha of 0.05 was set, with odds ratios of 0.68 and 0.54 for associations between leg fat and clustered cardiometabolic risk factors were assigned for male and female, respectively, and ORs of 1.63 and 1.34 for trunk fat in male and females, and ORs of 2.58 and 2.00 for android fat in males and females, respectively [33, 40]. As a result, at least 386 male participants and 664 female participants were required when the statistical power of 85% was reached.

**Fig. 1:**
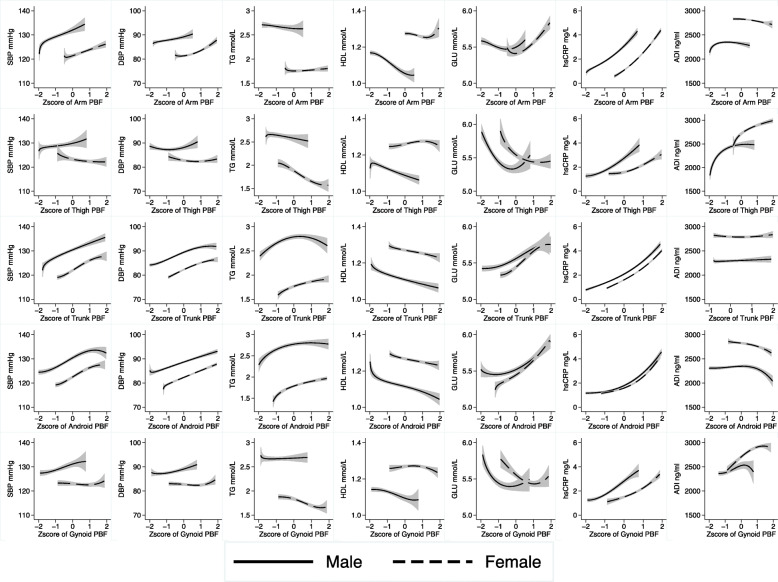
Association between regional PBF and continuous cardiometabolic indicators by sex. Lines represent means and areas represent 95%CI. Fractional polynomial regression analyses were performed to estimate the associations with adjustment for age, physical activity, smoking, alcohol drinking and the whole-body percentage of body fat (except for the association of whole body PBF). (PBF: percentage of body fat. SBP: systolic blood pressure. DBP: diastolic blood pressure. TG: triglyceride. HDL: High-density lipids cholesterol. GLU: Glucose. hsCRP: high sensitivity C-reactive protein. ADI: Adiponectin)

Firstly, the association between total/regional PBF and cardiometabolic indicators were assessed by linear regression models with adjusting for potential covariates (age, smoking, alcohol drinking, physical activities and whole body PBF). In addition, fractional polynomial regression models were performed to demonstrate the non-linear dose-response relationship between PBF and continuous cardiometabolic indicators (Fig. 1) [52–54].

Then, six continuous cardiometabolic variables were dichotomized for further analysis (high TG: ≥ 1.7 mmol/L, low HDL: < 1 mmol/L for male or < 1.3 mmol/L for female; elevated GLU: ≥ 5.6 mmol/ L, elevated BP: SBP ≥130 mmHg and/or DBP ≥ 85 mmHg, high hsCRP: > 30 mg/L; and low ADI: > 3.27 mg/L) [48–50]. Multivariate logistic regression models were used to estimate the associations between PBF and cardiometabolic risk factors with age, smoking, alcohol drinking, physical activities, and whole-body PBF adjusted in different sex groups. Also, the association between PBF and clustered cardiometabolic risk were analyzed with multivariate logistic regression models with potential covariates adjusted. Sensitivity analyses were conducted with a different definition of clustered cardiometabolic risk factors, defined as the presence of two or more of the five components of cardiometabolic risk (high TG, low HDL, elevated GLU, elevated BP, high hsCRP), which only included one inflammation-related indicator(high hsCRP but not low ADI) [55]. Also, we used MetS as the outcome as sensitivity analysis. Interaction effects between sex and regional PBF were investigated by including the interaction terms in the multivariate logistic regression models. All analyses were conducted using Stata (Version 14.0, Stata Corp, College Station, Texas) and SPSS for Windows (Version 20.0, SPSS Inc., Chicago, IL, USA).

## Results

A total of 1221 participants were included for analysis in this study, with a median age of 35 years and age range of 22–55 years. The participants’ demographic characteristics, regional PBF, and continuous variables of cardiometabolic metrics, lifestyle behavior variables and cardiometabolic risk factors were shown in Table 1. Female participants’ age was significantly higher than males (*P* < 0.001). Male participants have a significantly higher height, weight, BMI and WC than female participants (*P* < 0.001). Regarding the sex difference in body composition, female participants have a significant higher arm, thigh, trunk, android, gynoid and whole-body PBF than males (*P* < 0.001), with median differences of 14.2, 11.3, 4.9, 3.3, 11.6 and 7.7%, respectively. No significant difference in the time of sedentary behavior and physical activity level between male and female were observed (*P* > 0.05). Male participants have a significantly higher level of SBP, DBP, TG, lower HDL and lower ADI than females(*P* < 0.001), But the level of fasting glucose and hsCRP is not significantly different in males and females(*P* > 0.05). Males reported a higher rate of cigarette smoking and alcohol drinking than females(*P* < 0.001).

**Table 1:** General characteristics of the study population

Variables			Male	Female	Total	*P value*
Age(years)			33 (29,39)	37 (30,45)	35 (30,43)	< 0.001
Height(cm)			173 (169.1177.6)	159.4 (155.8163.5)	163.7 (158.1171.1)	< 0.001
Weight(kg)			88.4 (78.7,99.6)	71.9 (65.9,79.8)	77 (68.9,88.8)	< 0.001
WC(cm)			98.5 (93.2107)	91.1 (86,98.3)	94.2 (87.8101.7)	< 0.001
BMI(kg/m^2^)			29.4 (26.9,32.3)	28 (26.1,30.9)	28.5 (26.3,31.4)	< 0.001
Time of sedentary behavior(min)			360 (240,480)	330 (190,480)	360 (210,480)	0.161
Physical activity(MET-min/week)			1188 (495,2376)	1253 (594,2424)	1244 (570,2376)	0.696
Total and regional PBF	Arm PBF(%)		29.3 (25.8,32.8)	43.5 (40.6,46.9)	40.2 (31.6,44.8)	< 0.001
	Thigh PBF(%)		27.3 (24.6,30.7)	38.6 (35.3,41.4)	35.2 (28.7,39.7)	<0.001
	Trunk PBF(%)		40.4 (36.2,44)	45.3 (41.7,49)	43.5 (39.4,47.6)	< 0.001
	Android PBF(%)		43.9 (39.3,47.9)	47.2 (43.5,51.7)	46.1 (41.9,50.5)	< 0.001
	Gynoid PBF(%)		30.1 (27.1,33.6)	41.7 (38.4,44.7)	38.3 (32.2,43)	< 0.001
	Whole body PBF(%)		33.8 (30.9,36.8)	41.5 (38.8,44.5)	39.3 (34.9,42.9)	< 0.001
Continuous variables of cardiometabolic indicators	SBP (mmHg)		128 (120,138)	120 (112,129)	123 (115,133)	< 0.001
	DBP (mmHg)		88 (80,94)	81 (75,89)	83 (78,91)	< 0.001
	TG (mmol/L)		2.1 (1.49,2.91)	1.51 (1.19,1.99)	1.69 (1.27,2.36)	< 0.001
	HDL (mmol/L)		1.11 (0.99,1.23)	1.24 (1.09,1.4)	1.18 (1.04,1.34)	< 0.001
	GLU (mmol/L)		5.28 (4.93,5.71)	5.22 (4.89,5.68)	5.24 (4.91,5.69)	0.916
	hsCRP (mg/L)		1.03 (0.57,2.09)	1.13 (0.52,2.27)	1.08 (0.53,2.23)	0.277
	ADI (ug/ml)		2.04 (1.38,2.95)	2.65 (1.76,3.65)	2.43 (1.60,3.37)	< 0.001
Dichotomous variables of cardiometabolic risk factors	Cigarette Smoking	no	147 (46.7%)	547 (92.7%)	694 (76.7%)	< 0.001
		yes	168 (53.3%)	43 (7.3%)	211 (23.3%)	
	Alcohol drinking	no	163 (51.7%)	532 (90.2%)	695 (76.8%)	< 0.001
		yes	152 (48.3%)	58 (9.8%)	210 (23.2%)	
	MetS	no	170 (37.4%)	370 (48.3%)	540 (44.2%)	< 0.001
		yes	285 (62.6%)	396 (51.7%)	681 (55.8%)	
	Central obesity	no	19 (4.2%)	34 (4.4%)	53 (4.3%)	0.828
		yes	436 (95.8%)	732 (95.6%)	1168 (95.7%)	
	High TG	no	150 (33%)	465 (60.7%)	615 (50.4%)	< 0.001
		yes	305 (67%)	301 (39.3%)	606 (49.6%)	
	Low HDL	no	337 (74.1%)	299 (39%)	636 (52.1%)	< 0.001
		yes	118 (25.9%)	467 (61%)	585 (47.9%)	
	High glucose	no	317 (69.7%)	535 (69.8%)	852 (69.8%)	0.949
		yes	138 (30.3%)	231 (30.2%)	369 (30.2%)	
	High BP	no	157 (34.5%)	460 (60.1%)	617 (50.5%)	< 0.001
		yes	298 (65.5%)	306 (39.9%)	604 (49.5%)	
	high hsCRP	no	396 (87.2%)	626 (82.3%)	1022 (84.1%)	0.022
		yes	58 (12.8%)	135 (17.7%)	193 (15.9%)	
	low ADI	no	57 (18.3%)	188 (32.2%)	245 (27.3%)	< 0.001
		yes	255 (81.7%)	396 (67.8%)	651 (72.7%)	
	≥2 risk factors	no	41 (10.0%)	114 (16.9%)	115 (14.3%)	0.002
		yes	368 (90%)	561 (83.1%)	929 (85.7%)	

*WC* waist circumference, *BMI* body mass index, *PBF* percentage of body fat, *SBP* systolic blood pressure, *DBP* diastolic blood pressure, *TG* triglyceride, *HDL* High-density lipids cholesterol, *GLU* Glucose, *hsCRP* high sensitivity C-reactive protein, *ADI* Adiponectin, *BP* blood pressure, *MetS* Metabolic syndrome

The prevalence of MetS, central obesity, high TG, low HDL, high glucose, high BP, hsCRP and low ADI were 55.8, 95.7, 49.6, 47.9, 30.2, 49.5, 15.9 and 72.7%, respectively. Male participants have a higher prevalence of MetS, high TG, high BP and low ADI than female participants(*P* < 0.001). Female participants had a significantly higher risk of low HDL and higher hsCRP than males (*P* < 0.05). 85.7% of the participants have clustered cardiometabolic risk with significant sex difference (90% vs 83.1% for males and females, respectively).

Results of multivariate linear regression models were presented in Table 2 (Table 2). Both in males and females, Z score of WC was associated with higher TG, lower HDL, higher SBP, higher DBP and higher hsCRP(*P* < 0.05). In female participants, the Z score of WC is also associated with higher fasting glucose and lower ADI (*P* < 0.05), which is not statistically significantly in males(*P* > 0.05).

**Table 2:** Linear regression analysis between regional fat distribution and cardiometabolic indicators

Group	Cardiometabolic	Z score of WC		Z score of Arm PBF		Z score of Thigh PBF		Z score of Trunk PBF		Z score of Android PBF		Z score of Gynoid PBF		Z score of Whole Body PBF	
	Indicators	b(95%CI)	***P***	b(95%CI)	***P***	b(95%CI)	***P***	b(95%CI)	***P***	b(95%CI)	***P***	b(95%CI)	***P***	b(95%CI)	***P***
Male	TG, mmol/L	**0.56 (0.13, 0.99)**	**0.011**	− 0.47(− 1.36, 0.43)	0.303	**− 0.86(− 1.64, − 0.07)**	**0.033**	0.34(− 0.30, 0.98)	0.299	0.55(− 0.11, 1.21)	0.102	− 0.20(− 1.00, 0.60)	0.616	0.17(− 0.13, 0.47)	0.253
	HDL, mmol/L	**− 0.04(− 0.08,0.00)**	**0.034**	0.04(− 0.04, 0.11)	0.309	0.00(− 0.07, 0.06)	0.900	0.02(− 0.03, 0.07)	0.468	0.00(− 0.06, 0.06)	0.975	0.02(− 0.05, 0.09)	0.558	**− 0.05(− 0.07, − 0.02)**	**< 0.001**
	SBP, mmHg	**4.64 (1.83, 7.45)**	**0.001**	−3.84(− 9.7, 2.03)	0.199	**−8.70(− 13.8, − 3.60)**	**0.001**	1.49(− 2.70, 5.69)	0.484	2.38(− 1.95, 6.70)	0.281	**−6.13(− 11.34, − 0.93)**	**0.021**	**4.55 (2.59, 6.51)**	**< 0.001**
	DBP, mmHg	**3.67 (1.48, 5.86)**	**0.001**	− 4.04(− 8.61, 0.54)	0.083	**− 8.81(− 12.74, − 4.87)**	**< 0.001**	1.32(− 1.96, 4.60)	0.430	**3.49 (0.12, 6.85)**	**0.042**	**−6.56(− 10.6, − 2.53)**	**0.002**	**3.04 (1.51, 4.57)**	**< 0.001**
	GLU, mmol/L	0.19(− 0.02, 0.39)	0.077	0.16(− 0.26, 0.59)	0.458	− 0.36(− 0.74, 0.01)	0.057	0.00(− 0.31, 0.30)	0.996	− 0.01(− 0.32, 0.31)	0.972	**− 0.40(− 0.78, − 0.02)**	**0.037**	0.05(− 0.09, 0.19)	0.510
	hsCRP, mg/L	**1.09 (0.41, 1.77)**	**0.002**	**1.62 (0.20, 3.04)**	**0.025**	**−1.41(− 2.66, − 0.15)**	**0.028**	0.22(− 0.80, 1.25)	0.667	0.48(− 0.58, 1.53)	0.373	− 0.87(− 2.14, 0.41)	0.182	**1.13 (0.66, 1.61)**	**< 0.001**
	ADI, ug/ml	− 0.13(− 0.40, 0.14)	0.339	0.30(− 0.26, 0.85)	0.294	**0.78 (0.29, 1.27)**	**0.002**	− 0.34(− 0.73, 0.06)	0.096	− 0.38(− 0.79, 0.03)	0.066	**0.85 (0.36, 1.34)**	**0.001**	0.08(− 0.11, 0.26)	0.423
Female	TG, mmol/L	**0.23 (0.12, 0.34)**	**< 0.001**	−0.02(− 0.27, 0.22)	0.857	**− 0.58(− 0.75, − 0.42)**	**< 0.001**	**0.17 (0.01, 0.33)**	**0.039**	**0.38 (0.22, 0.54)**	**< 0.001**	**− 0.49(− 0.65, − 0.32)**	**< 0.001**	0.08(− 0.03, 0.19)	0.136
	HDL, mmol/L	**−0.07(− 0.10, − 0.04)**	**< 0.001**	0.05(− 0.01, 0.11)	0.122	**0.09 (0.05, 0.14)**	**< 0.001**	**−0.06(− 0.10, − 0.02)**	**0.004**	**−0.08(− 0.12, − 0.04)**	**< 0.001**	**0.06 (0.02, 0.11)**	**0.009**	−0.01(− 0.04, 0.01)	0.341
	SBP, mmHg	**4.57 (2.85, 6.29)**	**< 0.001**	0.77(− 3.11, 4.66)	0.696	**−6.17(− 8.87, − 3.48)**	**< 0.001**	**3.27 (0.71, 5.83)**	**0.012**	**3.15 (0.62, 5.68)**	**0.015**	**−4.65(− 7.36, − 1.93)**	**0.001**	**3.52 (1.85, 5.18)**	**< 0.001**
	DBP, mmHg	**3.70 (2.48, 4.91)**	**< 0.001**	0.56(− 2.20, 3.33)	0.688	**−5.26(−7.16, − 3.36)**	**< 0.001**	**3.44 (1.63, 5.24)**	**< 0.001**	**3.11 (1.32, 4.90)**	**0.001**	**−3.84(− 5.76, − 1.92)**	**< 0.001**	**3.00 (1.82, 4.19)**	**< 0.001**
	GLU, mmol/L	**0.35 (0.19, 0.51)**	**< 0.001**	−0.03(− 0.39, 0.33)	0.887	**− 0.69(− 0.93, − 0.44)**	**< 0.001**	***0.35 (0.12, 0.59)***	**0.004**	0.22(− 0.02, 0.45)	0.071	**− 0.61(− 0.86, − 0.36)**	**< 0.001**	**0.22 (0.06, 0.37)**	**0.006**
	hsCRP, mg/L	**0.59 (0.25, 0.93)**	**0.001**	0.29(− 0.46, 1.04)	0.449	− 0.53(− 1.05, 0.00)	0.051	0.30(− 0.19, 0.80)	0.230	− 0.19(− 0.68, 0.31)	0.457	−0.36(− 0.88, 0.17)	0.188	**1.01 (0.69, 1.34)**	**< 0.001**
	ADI, ug/ml	**−0.2(− 0.39, − 0.01)**	**0.039**	−0.13(− 0.55, 0.29)	0.541	**0.48 (0.19, 0.77)**	**0.001**	−0.23(− 0.5, 0.05)	0.104	**−0.29(− 0.56, − 0.02)**	**0.036**	**0.50 (0.21, 0.79)**	**0.001**	−0.01(− 0.18, 0.17)	0.960

Adjusted for age, physical activity, smoking, alcohol drinking and the whole body PBF (except for the association of whole body PBF). *WC* waist circumference, *PBF* percentage of body fat, *SBP* systolic blood pressure, *DBP* diastolic blood pressure, *TG* triglyceride, *HDL* High-density lipids cholesterol, *GLU* Glucose, *hsCRP* high sensitivity C-reactive protein, *ADI* Adiponectin. All significant results were marked in bold

For associations of regional PBF variables, the z score of arm PBF was significantly associated with hsCRP (β = 1.62, 95%CI: 0.20, 3.04) only in male participants. In both female and male participants, higher thigh PBF was associated with lower TG, lower SBP, lower DBP and higher ADI (*P* < 0.05). Higher thigh PBF was significantly associated with lower hsCRP (β = − 1.41, 95%CI: − 2.66, − 0.15, *P* = 0.028) in male participants only, and with higher HDL(β = 0.09, 95%CI: 0.05, 0.14, *P* < 0.001) and lower glucose(β = − 0.69, 95%CI: − 0.93, − 0.44, *P* < 0.001) in female participants only. Higher trunk PBF was significantly associated with higher SBP, higher DBP, higher TG, lower HDL, and higher glucose level in female participants only. In male participants, associations of trunk PBF and cardiometabolic indicators were not statistically significant. Higher android PBF was significantly associated with higher DBP in both female and male participants. But higher android PBF was significantly associated with higher TG, lower HDL, higher SBP, and lower ADI in female participants only. Higher gynoid PBF was significantly associated with lower SBP, lower DBP, lower glucose and higher ADI in both female and male participants. But higher gynoid PBF was significantly associated with lower TG and higher HDL in female participants only. Whole-body PBF was positively associated with SBP, DBP, glucose and hsCRP level in both females and males (*P* < 0.05). Whole-body PBF was positively associated with HDL in male participants only (*P* < 0.001). Whole-body PBF has positively associated with glucose in female participants only (*P* = 0.006).

In different sex groups, the non-linear relationships between regional measures of PBF and cardiometabolic metrics were demonstrated with adjustment of age, whole body PBF, physical activity, smoking and alcohol drinking (Fig. 1). Sex differences were observed in most of the associations between regional measures of PBF and cardiometabolic indicators, but similar patterns were observed in female and male participants in some associations, such as trunk PBF and hsCRP, trunk PBF and ADI, android PBF and hsCRP, android PBF and DBP. Although the direction of the associations is similar in female and male participants, but the effect sizes (measuring by slope) are quite different. For example, the line representing the association between TG and gynoid PBF is almost horizontal, while in female participants TG and gynoid PBF is negatively associated.

The association between z score of regional measures of PBF and risk of clustered cardiometabolic risk by sex was presented in Table 3. Significant sex difference in the associations between regional measures of PBF and clustered cardiometabolic risk were observed. In males, higher android and whole-body PBF was significantly associated with higher odds of clustered cardiometabolic risk. One-SD increase in android PBF is associated with a 3.39-folds of clustered cardiometabolic risk (95%CI: 1.42, 8.09). While in females, one SD increase of android PBF is associated with 2.91-folds of clustered cardiometabolic risk (95%CI: 1.75, 4.85). The ORs for whole-body PBF also differ by sex with OR of 2.45 (95%CI: 1.53–3.92) in males and 2.01 (95%CI: 1.47–2.76) in females. While trunk PBF was significantly associated with higher odds of clustered cardiometabolic risk with OR of 1.90 (95%CI:1.02–3.55) in females, but the association in males did not reach statistical significance (OR = 1.16, 95%: 0.42–3.19). Also, associations of regional measures of PBF and central obesity by sex were presented in Table S1. We also did sensitivity analysis with a different definition of clustered cardiometabolic risk, which only included one inflammation-related indicator (high hsCRP but not low ADI). Results of the sensitivity analysis were presented in Table S2, which showed similar associations. Also, similar results were observed for the associations between regional PBF and risk of MetS by sex(Table S3).

**Table 3:** Logistic regression between regional fat distribution and clustered cardiometabolic risk

Group	Fat distribution	Model 1		Model 2	
		OR (95% CI)	***P***	OR (95% CI)	***P***
Male	Arm PBF	**2.06 (1.10,3.86)**	**0.024**	0.70 (0.18,2.70)	0.608
	Thigh PBF	1.19 (0.69,2.05)	0.525	**0.06 (0.02,0.23)**	**< 0.001**
	Trunk PBF	**2.01 (1.37,2.94)**	**< 0.001**	1.16 (0.42,3.19)	0.772
	Android PBF	**2.38 (1.62,3.51)**	**< 0.001**	**3.39 (1.42,8.09)**	**0.006**
	Gynoid PBF	1.48 (0.88,2.50)	0.141	**0.17 (0.05,0.57)**	**0.004**
	Whole body PBF	**2.27 (1.43,3.63)**	**0.001**	**2.45 (1.53,3.92)**	**< 0.001**
Female	Arm PBF	**2.33 (1.57,3.47)**	**< 0.001**	1.51 (0.75,3.07)	0.251
	Thigh PBF	0.92 (0.67,1.26)	0.596	**0.20 (0.12,0.35)**	**< 0.001**
	Trunk PBF	**1.92 (1.48,2.49)**	**< 0.001**	**1.90 (1.02,3.55)**	**0.043**
	Android PBF	**2.09 (1.63,2.68)**	**< 0.001**	**2.91 (1.75,4.85)**	**< 0.001**
	Gynoid PBF	1.06 (0.77,1.46)	0.732	**0.22 (0.12,0.41)**	**< 0.001**
	Whole body PBF	**2.00 (1.47,2.74)**	**< 0.001**	**2.01 (1.47,2.76)**	**< 0.001**

Model 1: crude model without adjusting any covariates. Model 2: adjusted for age, physical activity, smoking, alcohol drinking and the whole body PBF (except for the association of whole body PBF). PBF: percentage of body fat. Clustered cardiometabolic risk was defined as presence of two or more than two risk factors of the six components of cardiometabolic risk (high triglyceride, low high-density lipids cholesterol, elevated glucose, elevated blood pressure, elevated high sensitivity C-reactive protein, and low adiponectin). All significant results were marked in bold

In male participants, with adjustment of potential covariates, higher thigh and gynoid adiposity were significantly associated with a decreased odd of clustered cardiometabolic risk with OR of 0.06 (95%CI: 0.02–0.23) and 0.17 (95%CI: 0.05–0.57), respectively. While in females, thigh and gynoid PBF were significantly associated with less low odds of clustered cardiometabolic risk with OR of 0.20 (95%CI: 0.12–0.35) and 0.22(95%CI: 0.12–0.41), respectively.

**Fig. 2:**
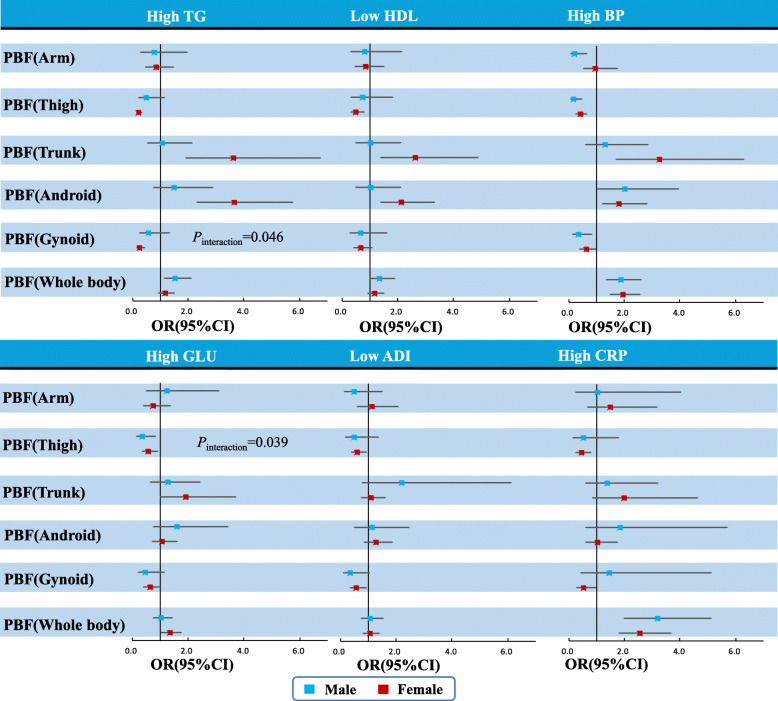
OR (95%CI) for Cardiometabolic risk factors associated with a 1-SD increase in percentage of regional body fat (PBF), stratified by sex (adjusted for age, physical activity, smoking, alcohol drinking and the whole-body PBF [except for the association of whole-body PBF]). PBF: percentage of body fat. BP: blood pressure. TG: triglyceride. HDL: High-density lipids cholesterol. GLU: Glucose. hsCRP: high sensitivity C-reactive protein. ADI: Adiponectin

Estimated relationships between regional measures of PBF and risk of six specific cardiometabolic risk factors (high TG, low HDL, elevated GLU, elevated BP, high hsCRP, and low ADI) were shown from the forest plots (Fig. 2). In females, trunk and android PBF were related to a significant increase of high TG, and thigh or gynoid PBF were related to the decrease of high TG. Similar sex-specific results were found for low HDL. A significant interaction between sex and gynoid PBF on the risk of high TG was observed (*P*_*interaction*_ = 0.046, Fig. 2). In female participants, trunk, android, and whole-body PBF were related to the significant increase of high BP, while in male participants, the lower body PBF (thigh and gynoid) and arm PBF were favorable for high BP. In male participants, thigh PBF was a protective factor for abnormal glucose. Both thigh and gynoid PBF were protective factors for abnormal glucose in female participants, whereas trunk PBF was a risk factor for abnormal glucose. Nevertheless, there was a significant interaction between sex and thigh PBF on the risk of abnormal glucose (*P*_*interaction*_ = 0.039, Fig. 2). Thigh and gynoid PBF were protective factors for high hsCRP in female participants only. Thigh and gynoid PBF were protective factors for low ADI in female participants, and in male participants only gynoid PBF was the protective factor for low ADI.

## Discussion

In this cross-sectional study with 455 males and 766 females with overweight or obesity, we found adiposity distribution is strongly associated with cardiometabolic risk factors and clustered cardiometabolic risk. Significant quantitative differences by sex were observed in those associations. In a word, for both men and women central obesity (android PBF or WC) could be the best anthropometric measurement for screening people at risk for CVD risk factors. Upper body fat is more strongly associated with higher odds of clustered cardiometabolic risk in women than men, whereas lower body fat is more protective in men than women. A significant association between fat accumulation in the trunk area and clustered cardiometabolic risk was observed in females only. Significant interactions between sex and thigh PBF or gynoid PBF were identified. These findings suggest that regional adiposity distribution in arm, thigh, trunk, android and gynoid areas have effects on cardiometabolic indicators with significant quantitative sex difference.

Similar with previous studies in Chinese children [40] or other population [56], central obesity (measured by android fat) is the strongest anthropometric measure for screening clustering of cardiometabolic risk factors independent of other potential covariates. Numerous previous studies have showed that abdominal fat accumulation measured by waist circumference is significantly associated with higher cardiometabolic risk [57–59]. Our study used a more precise central obesity measure (by android PBF) to confirm and extend prior findings and further explored its sex difference. The findings indicate that fat accumulation in the abdomen plays an important role in the development or progression of clustering of cardiometabolic risk factors in young overweight or obese individuals.

Consistent with studies of other population including Chinese, the present study confirmed the protective effects of the thigh and gynoid fat on cardiometabolic health in both female and males with overweight or obesity [33, 40, 60]. Additionally, our study showed that the associations between thigh or gynoid fat and clustered cardiometabolic risk were more profound in men than in women. The observed sex differences may be explained by varied hormones effect and the ratio of visceral and subcutaneous fat mass in the lower and upper body. Date back to prenatal life, which is the “metabolic programming period”, sex hormones have organizational effects on the body composition and metabolism [61]. After puberty, sex hormones have activation effects on glucose and energy homeostasis [61]. On the other hand, men are likely to have higher visceral fat and less subcutaneous fat in the upper body than women with the same level of total fat mass [22]. It is believed that visceral fat, other than subcutaneous fat, is a strong predictor of incident metabolic syndrome [23]. Nevertheless, future studies are warranted to explore the specific underlying mechanism for the sex-related difference of associations between the thigh and gynoid fat with cardiometabolic health. Findings may be crucial to support sex-specific evaluation and intervention framework.

Researchers made a hypothesis that lower body adiposity may act as a metabolic buffer of dietary fat or lipids to protect other tissues from lipotoxicity, which results from ectopic fat deposition and lipid overflow [62]. Likewise, high leg or thigh adiposity is related to a significantly higher level of HDL (high-density lipoprotein cholesterol), suggesting a close relation between lower-body adiposity’s TG (triglyceride) clearance capacity and a favorable lipid profile [63].

We identified protective effects of the thigh and gynoid adiposity on abnormal glucose in female participants in this study, which is consistent with previously published studies [45, 64]. The Snijder’s study in a Dutch adult population [45] and the Quebec Family Study [65] also identified that fat in the legs is protective for the abnormal glucose metabolism. It was well documented that subcutaneous fat in the thigh is beneficial for cardiometabolic health, including glucose metabolism [62, 66]. The sex difference of the protective effect could be explained by the ratio of subcutaneous fat in the thighs, which is more than 80% in men and > 90% in women [67].

However, a much lower proportion of subcutaneous fat and a higher ratio of visceral fat were found in abdominal fat, while visceral fat is proved to be detrimental for the cardiometabolic profile [62, 67]. In this study, we confirmed the detrimental effect of the trunk and android fat on clustered cardiometabolic risk, which were reported in previous studies [39, 45, 60]. Further, we identified that the unfavorable effect of trunk fat on clustered cardiometabolic risk is significant in female participants only. We found a smaller OR in males than that in females, and the association in males is not statistically significant. Therefore, the link between trunk fat and clustered cardiometabolic risk is profound in females, which is consistent with previous findings [45].

Furthermore, this study examined associations between inflammatory markers and regional adiposity. It’s well known that obesity is usually accompanied by a low or moderate grade of inflammation [9]. we found that thigh and gynoid PBF were protective for high hsCRP only in female participants. Similar to this, Wu et al. reported the favorable effect of leg fat mass with inflammatory markers (CRP and IL-6) in both sex groups [33]. Another American study also showed that the distribution and quantity of fat impact the CRP level to a greater extent in females than in males [68]. The possible sex difference in the association between fat distribution and inflammatory level might be mediated by sex hormones because estrogen could significantly raise the CRP level in females [69].

For the anti-inflammatory adipokine, adiponectin, in female participants, thigh and gynoid PBF is protective for low ADI, while in male participants only gynoid is protective for low ADI. The beneficial effect of the thigh or gynoid adiposity for low adiponectin is consistent with previous studies [33, 70, 71]. Adiponectin has anti-inflammatory, insulin-sensitizing, and anti-atherosclerotic effect [70, 72, 73]. One possible explanation for the protective effect of the thigh and android fat on adiponectin could be the difference in adiponectin secretion rate in different fat depots [70]. There is evidence showing that the secretion rate of adiponectin in cells from intra-abdominal adipocytes is significantly different from subcutaneous adipocytes [74], but the difference in the adiponectin secretion rate in adipocytes in the thigh or gynoid fat tissue is not clear now.

The accurate quantification of regional body fat by the DXA method is one of the strengths of this study. Additionally, with relatively large sample size, we have controlled for a list of potential covariates, including age, physical activity, smoking, alcohol drinking and the whole-body percentage of body fat. Yet, there are several limitations in the present study. Firstly, this is a cross-sectional study, we could not conclude in regards to the causality or directionality of the associations between regional fat distribution and cardiometabolic risk factors. Secondly, participants of this study included overweight and obese Chinese young adults with a median age of 35 years old. The findings may not be representative of other population. Future studies in normal-weight young adults or other population would be important to add information on the associations between adiposity distribution and cardiometabolic health. Thirdly, as this is a secondary analysis of data collected for other purposes, potential confounders may have not been included in the models, such as the use of hormonal drugs.

## Conclusion

This study showed that there are opposite associations between upper body fat (trunk, and android fat) or lower body fat (thigh and gynoid fat) with clustered cardiometabolic risk irrespective of the total body fat. Generally, central obesity (measured by android PBF or WC) could be the best anthropometric measurement for screening people at risk for CVD risk factors for both men and women. In addition, sex differences in the associations were found, upper body adiposity is more detrimental to cardiometabolic health in women than men, while lower body fat is relatively protective in men more than women. In future interventional studies or risk stratification research on cardiometabolic health, fat distribution and its sex-related different association with cardiometabolic risk should be considered, to obtain the most beneficial outcomes in cardiometabolic health for both women and men. Future studies should be warranted to explore the contribution of sex hormones to the association between adiposity distribution and cardiometabolic health.

## Supplementary Information

**Additional file 1: Table S1.** Logistic regression between central obesity and cardiometabolic risk factors.

**Additional file 2: Table S2.** Logistic regression between regional fat distribution and clustered cardiometabolic risk with another definition*.

**Additional file 3: Table S3.** Logistic regression between regional fat distribution and MetS.

## Data Availability

The datasets collected and analyzed in our study are available on reasonable request from the first author or corresponding author (Yide Yang, Email: yangyide2007@126.com, and Bin Dong, email: bindong@bjmu.edu.cn).

## Abbreviations

hsCRP: High sensitivity C-reactive protein
ADI: Adiponectin
MetS: Metabolic syndrome
SBP: Systolic blood pressure
DBP: Diastolic blood pressure
TG: Triglycerides
TC: Total cholesterol
HDL: High density lipoprotein cholesterol
LDL: Low density lipoprotein cholesterol
MET: Metabolic equivalent
