# Supplementary material for: Sex differences in the associations between adiposity distribution and cardiometabolic risk factors in overweight or obese individuals: a cross-sectional study
Source: BMC Public Health. 2021 Jun 26;21:1232. doi: 10.1186/s12889-021-11316-4 (PMC8234731; doi:10.1186/s12889-021-11316-4)
Supplement: Supplementary file 1 — Additional file 1: Table S1. Logistic regression between central obesity and cardiometabolic risk factors. [file 12889_2021_11316_MOESM1_ESM.docx]

| **Table S1. Logistic regression between central obesity and cardiometabolic risk factors.** | | | | | | | |
| --- | --- | --- | --- | --- | --- | --- | --- |
| **Group** | **Risk factors** | **Model 1** | | | **Model 2** | | |
|  |  | **OR** | **95% CI** | ***P*** | **OR** | **95% CI** | ***P*** |
| **Male** | **high TG** | 1.88 | (0.75,4.74) | 0.179 | **3.96** | **(1.14,13.71)** | **0.030** |
|  | **low HDL** | 1.91 | (0.55,6.68) | 0.311 | 3.73 | (0.47,29.70) | 0.214 |
|  | **high BP** | **3.44** | **(1.33,8.92)** | **0.011** | **3.56** | **(1.03,12.31)** | **0.045** |
|  | **elevated GLU** | 1.23 | (0.43,3.48) | 0.698 | 2.03 | (0.42,9.85) | 0.379 |
|  | **high hsCRP** | 2.71 | (0.36,20.73) | 0.336 | 1.40 | (0.17,11.37) | 0.753 |
|  | **low ADI** | - | - | - | - | - | - |
|  | **≥2 risk factors** | **7.57** | **(2.31,24.77)** | **0.001** | **6.43** | **(1.91,21.60)** | **0.003** |
| **Female** | **high TG** | 2.17 | (0.97,4.86) | 0.060 | 2.09 | (0.90,4.84) | 0.087 |
|  | **low HDL** | **2.05** | **(1.02,4.09)** | **0.043** | **2.71** | **(1.24,5.94)** | **0.012** |
|  | **high BP** | **2.66** | **(1.15,6.20)** | **0.023** | **2.88** | **(1.13,7.32)** | **0.027** |
|  | **elevated GLU** | 2.07 | (0.85,5.07) | 0.111 | 2.31 | (0.86,6.20) | 0.098 |
|  | **high hsCRP** | 3.58 | (0.85,15.13) | 0.083 | 2.75 | (0.64,11.81) | 0.175 |
|  | **low ADI** | 1.52 | (0.71,3.25) | 0.280 | 1.57 | (0.73,3.38) | 0.248 |
|  | **≥2 risk factors** | **3.11** | **(1.44,6.71)** | **0.004** | **3.13** | **(1.44,6.80)** | **0.004** |
| Model 1: crude model without adjusting any covariates. Model 2: adjusted for age, physical activity, smoking, and alcohol drinking. All significant results were marked in bold. SBP: systolic blood pressure. DBP: diastolic blood pressure. TG: triglyceride. HDL: High-density lipids cholesterol. GLU: Glucose. hsCRP: high sensitivity C-reactive protein. ADI: Adiponectin. | | | | | | | |
