# Supplementary material for: Sex differences in the associations between adiposity distribution and cardiometabolic risk factors in overweight or obese individuals: a cross-sectional study
Source: BMC Public Health. 2021 Jun 26;21:1232. doi: 10.1186/s12889-021-11316-4 (PMC8234731; doi:10.1186/s12889-021-11316-4)
Supplement: Supplementary file 2 — Additional file 2: Table S2. Logistic regression between regional fat distribution and clustered cardiometabolic risk with another definition*. [file 12889_2021_11316_MOESM2_ESM.docx]

| **Table S2. Logistic regression between regional fat distributions and clustered cardiometabolic risk with another definition*.** | | | | | |
| --- | --- | --- | --- | --- | --- |
| Group | Fat distribution | Model 1 | | Model 2 | |
|  |  | OR (95% CI) | *P* | OR | *P* |
| Male | Arm PBF | 8.46(2.96,24.18) | <0.001 | 0.07(0.01,0.96) | 0.047 |
|  | Thigh PBF | 6.31(2.35,16.98) | <0.001 | 0.03(0.00,0.48) | 0.014 |
|  | Trunk PBF | 4.11(2.28,7.42) | <0.001 | 0.26(0.11,0.65) | 0.004 |
|  | Android PBF | 8.80(4.02,19.26) | <0.001 | 20.14(3.51,115.7) | 0.001 |
|  | Gynoid PBF | 5.86(2.33,14.70) | <0.001 | 0.04(0.00,0.52) | 0.014 |
|  | Whole body PBF | 11.59(4.75,28.25) | <0.001 | 12.85(4.11,40.2) | <0.001 |
| Female | Arm PBF | 3.30(1.74,6.27) | <0.001 | 0.17(0.04,0.73) | 0.017 |
|  | Thigh PBF | 1.31(0.78,2.21) | 0.311 | 0.07(0.02,0.19) | <0.001 |
|  | Trunk PBF | 4.32(2.72,6.86) | <0.001 | 2.13(1.26,3.60) | 0.005 |
|  | Android PBF | 4.72(3.02,7.38) | <0.001 | 4.51(2.13,9.55) | <0.001 |
|  | Gynoid PBF | 1.58(0.96,2.61) | 0.072 | 0.04(0.01,0.14) | <0.001 |
|  | Whole body PBF | 5.13(2.96,8.90) | <0.001 | 4.62(2.48,8.61) | <0.001 |
| Model 1: crude model without adjusting any covariates. Model 2: adjusted for age, sex, physical activity, smoke, alcohol drinking and the whole body PBF (except for the association of whole body PBF). PBF: percentage of body fat. *Clustered cardiometabolic risk was defined as the presence of two or more risk factors of the five components of cardiometabolic risk (high TG, low HDL, elevated GLU, elevated BP, and high hsCRP). | | | | | |
