# Supplementary material for: Sex differences in the associations between adiposity distribution and cardiometabolic risk factors in overweight or obese individuals: a cross-sectional study
Source: BMC Public Health. 2021 Jun 26;21:1232. doi: 10.1186/s12889-021-11316-4 (PMC8234731; doi:10.1186/s12889-021-11316-4)
Supplement: Supplementary file 3 — Additional file 3: Table S3. Logistic regression between regional fat distribution and MetS. [file 12889_2021_11316_MOESM3_ESM.docx]

|  |  |
| --- | --- |

| **Table S3. Logistic regression between regional fat distribution and MetS.** | | | | | |  |
| --- | --- | --- | --- | --- | --- | --- |
| **Group** | **Fat distribution** | **Model 1** | | **Model 2** | |  |
|  |  | **OR (95% CI)** | ***P*** | **OR (95% CI)** | ***P*** |  |
| Male | Arm PBF | 1.27(0.91,1.75) | 0.158 | **0.33(0.12,0.88)** | **0.026** |  |
|  | Thigh PBF | 1.20(0.89,1.61) | 0.236 | **0.29(0.13,0.69)** | **0.005** |  |
|  | Trunk PBF | **1.62(1.31,2.02)** | **<0.001** | 1.09(0.56,2.10) | 0.809 |  |
|  | Android PBF | **1.62(1.32,1.99)** | **<0.001** | 1.58(0.82,3.04) | 0.170 |  |
|  | Gynoid PBF | 1.32(0.99,1.76) | 0.059 | **0.39(0.17,0.90)** | **0.026** |  |
|  | Whole body PBF | **1.63(1.28,2.08)** | **<0.001** | **1.81(1.32,2.48)** | **<0.001** |  |
| Female | Arm PBF | **1.40(1.08,1.8)** | **0.010** | 0.83(0.47,1.45) | 0.504 |  |
|  | Thigh PBF | 0.81(0.65,1.01) | 0.061 | **0.28(0.18,0.43)** | **<0.001** |  |
|  | Trunk PBF | **1.88(1.57,2.25)** | **<0.001** | **6.52(3.40,12.49)** | **<0.001** |  |
|  | Android PBF | **1.82(1.53,2.15)** | **<0.001** | **2.99(1.92,4.64)** | **<0.001** |  |
|  | Gynoid PBF | 0.94(0.75,1.17) | 0.569 | **0.35(0.22,0.55)** | **<0.001** |  |
|  | Whole body PBF | **1.59(1.30,1.94)** | **<0.001** | **1.69(1.32,2.16)** | **<0.001** |  |
| Model 1: crude model without adjusting any covariates. Model 2: adjusted for age, physical activity, smoking, alcohol drinking and the whole body PBF (except for the association of whole body PBF). PBF: percentage of body fat. MetS: metabolic syndrome). All significant results were marked in bold. | | | | | |  |
|  |  |  |  |  |  |  |
